# Genetically engineered eye-colonizing microbes that deliver the anti-inflammatory cytokine interleukin-10 enhance corneal tissue repair

**DOI:** 10.1016/j.celrep.2026.117064

**Published:** 2026-03-05

**Authors:** Jackie Shane, Matthew Evans, Yannis Rigas, Robert M.Q. Shanks, Anthony J. St. Leger

**Affiliations:** 1 Department of Ophthalmology, University of Pittsburgh, Pittsburgh, PA 15219, USA; 2 Department of Immunology, University of Pittsburgh, Pittsburgh, PA 15213, USA; 3 Lead contact

## Abstract

Barrier surfaces harbor tissue-colonizing microbes that can shape local physiology and immunity. During corneal injury, inflammation can delay healing, resulting in loss of visual acuity. Standards of care include topical applications of therapies, which are quickly washed away, requiring a laborious treatment regimen to maintain efficacy. To address this problem, we engineered an eye-colonizing microbe, *Corynebacterium mastitidis*, to act as a long-term therapeutic delivery vehicle by secreting bioactive interleukin (IL)-10 using a native secretion signal that we identified using transposon mutagenesis. Engineered microbes stably colonize the eye and release mouse (mIL-10) or human IL-10 (hIL-10) that modulates local immunity and accelerates wound repair after an initial inoculation event. Further, hIL-10 producing *C. mast* can regulate inflammatory cytokine production in immune cells, highlighting the immune-regulatory capabilities of this live biotherapeutic product. These findings demonstrate that genetically engineered eye-colonizing bacteria can serve as a self-sustaining therapeutic platform to control inflammation and promote tissue repair.

## In brief

Engineered bacteria provide a platform for localized delivery of therapeutics. Here, Shane et al. show that an engineered eye commensal can accelerate corneal wound healing by promoting epithelial wound closure and regulating inflammation. These data highlight the utility of using bacteria as a live biotherapeutic to treat ocular surface disease.

## Graphical Abstract

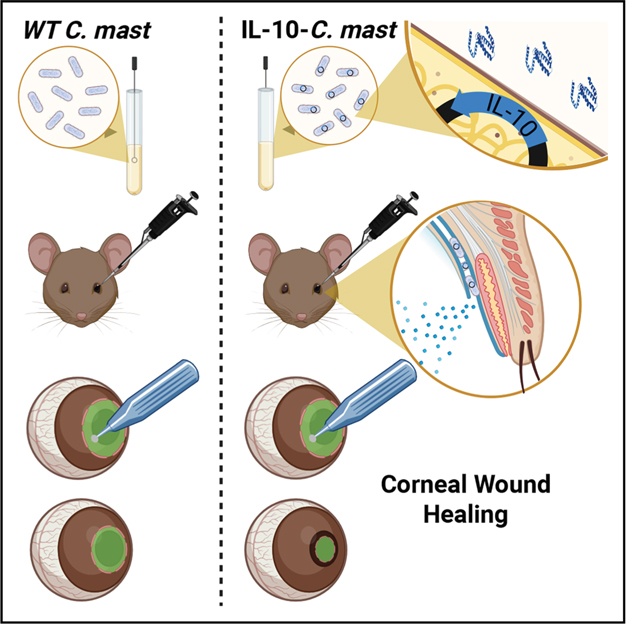

## INTRODUCTION

Corneal wounds remain a serious concern for ocular surface health, as over 1 million Americans are afflicted with corneal wounds annually and account for 3% of all visits to the emergency room.^1^ During wound healing, the eye is susceptible to infection and inflammatory damage, which can lead to other, more serious issues such as nerve damage,^2,3^ opacification, and loss of visual acuity.^4,5^ Therefore, increasing the rate of wound healing is paramount in preventing long-term disease.

Corneal wound healing relies on an appropriately mounted immune response that encourages healing and limits inflammation. After corneal wound healing, the upregulation of inflammatory cytokines, interleukin (IL)-1b, IL-6, and TNF-α, suppresses STAT3 activity, which can impair the proliferation of corneal epithelium and disrupt normal healing of the cornea after injury.^6,7^ Alternatively, IL-17 from cornea-infiltrating γδ T cells can activate STAT3, which enhances wound healing.^8,9^ Similarly, IL-10 can also activate STAT3 to encourage proliferation and migration of epithelial cells both in the cornea^7^ and other barrier surfaces throughout the body.^7,10,11^ In regard to the host immune system, IL-10 directly inhibits inflammation by suppressing cytokine production and proliferation of inflammatory myeloid cells. Furthermore, IL-10 interferes with the activation of T cells that require CD28 co-stimulation^12–14^ and suppresses T cells that exacerbate inflammation in many ocular surface diseases, such as infectious keratitis and dry eye disease^2,15,16^

Virtually all barrier sites have a resident microbiome that interacts with the host and fosters tissue barrier function by tuning host immune responses.^17^ Microbial members of the microbiome have adapted mechanisms that allow them to thrive at the sites of colonization. These unique adaptations make them attractive candidates to continually deliver naturally derived therapeutics to sites of interest. Indeed, scientists have begun engineering components of the intestinal microbiome to modulate metabolism^18^ as well as cytokine production to regulate immunopathology associated with mouse models of colitis.^19–21^ Further, clinical trials are also underway using IL-10-expressing bacteria as a therapeutic for Crohn’s disease.^22^ When IL-10 was expressed by *Lactococcus lactis*, a gut bacterium, CD4^+^ T cell-mediated colitis was reduced^21^; however, the direct effects of mouse IL-10 (mIL-10) signaling in this model are still unclear. On the ocular surface, therapeutics are continually washed away or diluted by the flow of tears^4,23^ and therefore require multiple laborious applications. In fact, older populations self-report forgetting their eyedrops 40% of the time^24^ and only 10% adherence when following the specified frequency of application for multiple applications per day.^25^ Therefore, engineering eye-colonizing bacteria that can deliver therapeutics to the ocular surface may provide a useful alternative to reduce the burden of applying therapies such as eye drops multiple times per day.

Recently, attention has been directed to host-microbiome interactions at low-biomass sites such as the lungs, skin, and eye and how those microbes may modulate physiology. Identifying relevant and tissue-colonizing bacteria has been difficult at these sites due to their paucibacterial nature; however, an eye-colonizing bacterium, *Corynebacterium mastitidis* (*C. mast*), has been isolated and cultured from the mouse conjunctiva. While *C. mast* elicits protective immune responses at the ocular surface, colonization of immunocompetent hosts does not result in clinically observable inflammation or pathology. Therefore, *C. mast* is an attractive target for engineering, as it appears non-pathogenic during homeostasis.^26^

Many factors must be considered in the development of an ocular live biotherapeutic product (LBP). With a few exceptions, including *Bacillus subtilis*, Gram-positive bacteria can be difficult to manipulate genetically. Until recently, there was no established set of tools for expressing or manipulating genes in *C. mast*. In this study, we employed a method of transposon mutagenesis, which has previously resulted in successful genetic manipulation of *C. mast*.^27^ Another consideration for the development of an LBP is that the therapeutic of interest must be secreted from the bacterium so that it can interact with surrounding host cells. Therefore, a secretion signal that is unique for the selected bacterium, *C. mast*, is essential for the proper development of a physiologically relevant ocular LBP. A final consideration for these studies is retaining the ability to colonize the ocular surface after genetic manipulation. This allows for the continual delivery of the therapeutic to the tissue of interest. Given the beneficial immune regulatory and tissue repair characteristics associated with IL-10 signaling, we engineered *C. mast* to produce and secrete IL-10 while maintaining the machinery required to colonize the eye.

In this study, we have identified a secretion signal from *C. mast* using a phosphatase transposon mutant library. After incorporating the secretion signal upstream of the gene for mIL-10 into a transposon cassette, we introduced the gene cassette into the *C. mast* genome by transposition. Multiple candidates produced and secreted mIL-10 *in vitro* and *in vivo*, which led to enhanced wound healing when mIL-10-*C. mast* colonized the eye. Further, we optimized this LBP to secrete human IL-10 (hIL-10) and showed that it could enhance *in vitro* wound healing in human cells and *in vivo* in mice. Together, these data demonstrate proof of concept that an ocular bacterium can be genetically engineered to act as an LBP to improve corneal wound healing, and we further show the clinical relevance and potential of this technology to reach patients in the future by incorporating the human form of IL-10.

## RESULTS

### Identification of native *C. mast*-specific secretion signals

**Figure 1.**
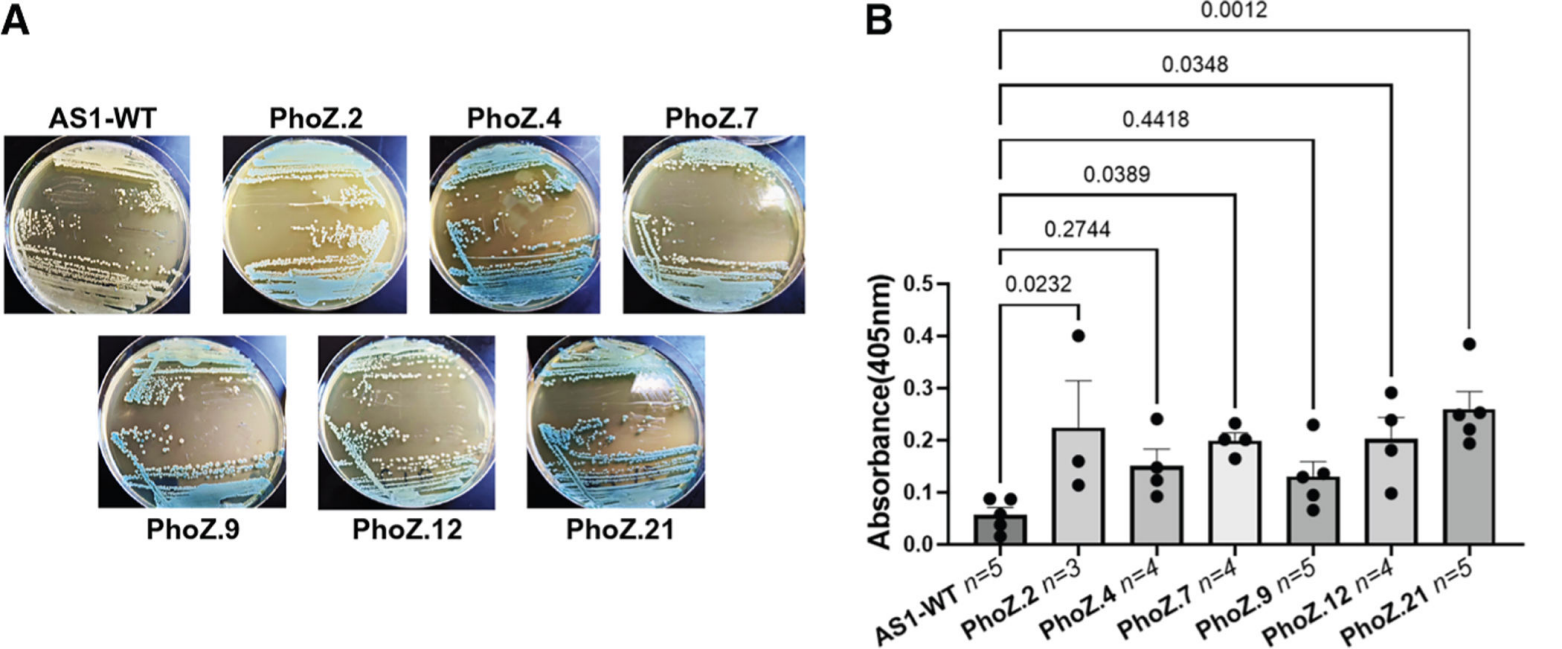
Identification of native *C. mast*-specific secretion signals (A) Representative images of PhoZ-secreting *C. mast* mutants created using EZ-TN5 transposase and a phosphatase/kanamycin resistance transposon gene cassette. Six transposon mutants analyzed from the library were able to significantly secrete phosphatase outside the cell, demonstrated by colorimetric change on agar supplemented with 5-bromo-4-chloro-3-indolyl phosphate (BCIP), repeated in triplicate. (B) Absorbances after p-nitrophenyl phosphate (PNPP) were quantified across all six isolates and WT control. The bars represent the mean absorbance ± SEM. The experiment was performed at least 3 independent times, and the dots represent the averages of technical duplicates used for each isolate. Significance was determined using an ANOVA and compared to the WT *C. mast* (AS1) control.

Bacteria use secretion signals to release proteins into the extracellular milieu. These secretion signals can be highly specialized and specific to the bacterium of interest.^28^ Because *C. mast* secretion signals are currently unknown, we created a phosphatase (*PhoZ*) transposon *C. mast* library to identify *C. mast*-specific secretion signals. We used transposon mutagenesis to incorporate a gene cassette containing kanamycin resistance and the *phoZ* reporter gene into the *C. mast* genome. When grown on agar plates supplemented with 5-bromo-4-chloro-3-indolyl phosphate (BCIP), a colorimetric substrate for PhoZ, mutant colonies that actively secrete PhoZ appear blue because BCIP cannot penetrate the cell membrane (Figure 1A). Secretion of PhoZ by positive transformants was quantified by growing mutants in liquid media to log phase and by analyzing phosphatase activity in the supernatant using a p-nitrophenyl phosphate (PNPP) assay (Figure 1B). DNA from the four isolates with a significantly greater concentration of secreted protein was purified for whole-genome sequencing to identify *C. mast* genes containing the transposon insert. We found that the isolate with the highest secretion activity, isolate 21, had the transposon insert within a predicted surface-anchored protein (NCBI Reference Sequence: WP_157824337.1). Using the SignalP 5.0^29^ online software, we isolated the signal sequence and characterized it as a Sec/signal peptidase I (SpI); this signal peptide was used to make the mIL-10 transposon.

### *C. mast* mutants can secrete functional mIL-10 that induces immunological changes in T cell culture

**Figure 2.**
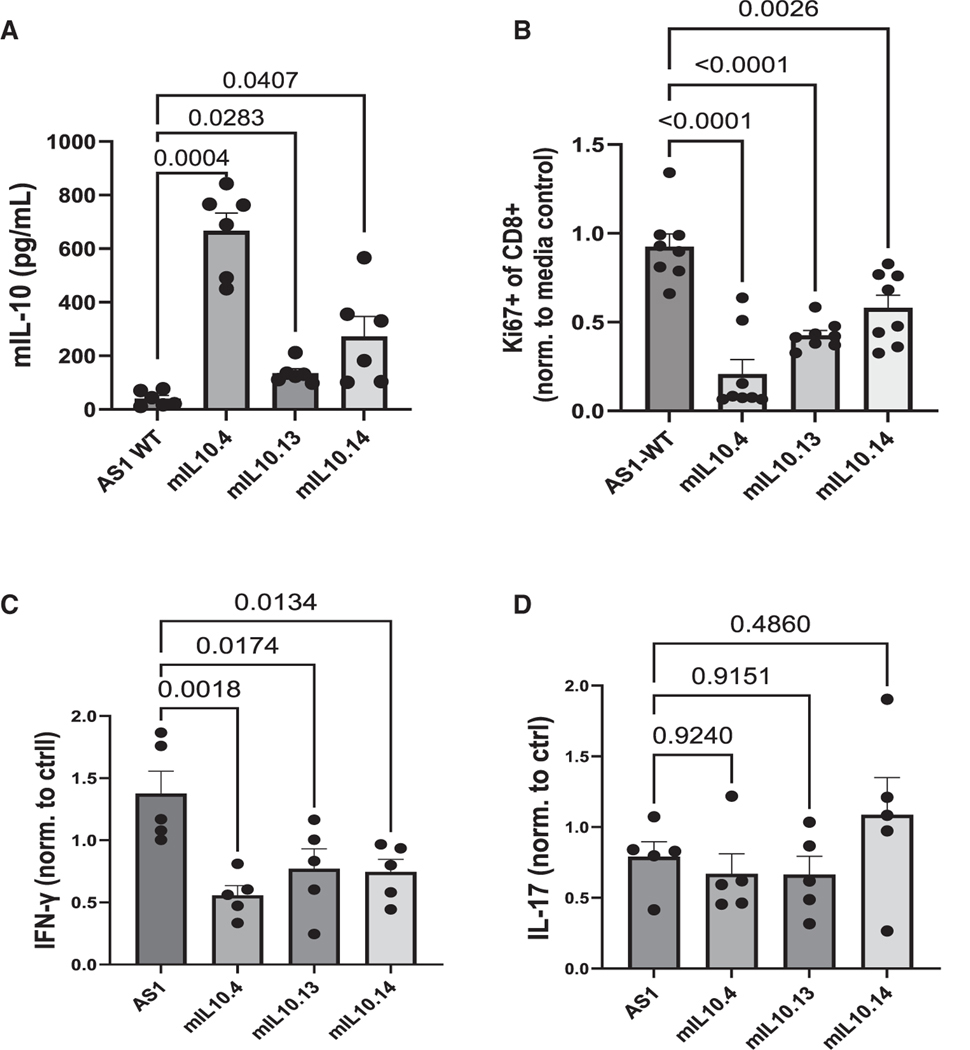
*C. mast* mutants can secrete functional mIL-10 that induces immunological changes in T cell culture (A) Control and transposon mutant bacteria were grown to log phase, and each culture was pelleted. Supernatants from bacterial cultures were assessed by ELISA. Concentrations were extrapolated from absorbances acquired from the IL-10 standard curve. The bars represent the mean IL-10 concentration ± SEM, and the dots represent the average of two technical replicates. Data were pooled from three independent experiments. (B–D) Memory (CD44^hi^CD62L^low^) T cells (αβ and γδ T cells) were isolated by fluorescence-activated cell sorting (FACS), labeled with CellTrace Violet dye, and stimulated with plate-bound αCD3 and soluble CD28 for 4 days. Bacteria were grown to log phase, and supernatants from AS1 or the mIL-10 transposon mutants were added to cultures after 24 h. (B) Proliferation was measured by flow cytometry as determined by a loss of CellTrace dye. Data were normalized to the media control. The bars represent the mean fold change in proliferation ± SEM. Data were pooled from three independent experiments. (C and D) Four days after the initiation of stimulation, brefeldin A was added to stimulated T cells, and cytokines were assessed by flow cytometry. Data were normalized to the media control. The bars represent the mean fold change in (C) IFN-γ or (D) IL-17 production ± SEM. Data were pooled from five independent experiments, and the dots represent the average of three technical replicates. *p* values were determined using an ANOVA.

We generated a gene cassette that consisted of the secretion signal from PhoZ.21 upstream of a codon-harmonized mIL-10 and an antibiotic resistance gene. To incorporate this cassette into the *C. mast* genome, we used transposase to generate a mIL-10 transposon mutant library according to our previous study.^27^ We found over 50 isolates that secreted ELISA-detectable mIL-10; these were collected, and the three best candidates are shown in Figure 2A. These data suggested that *C. mast* properly produced and secreted mIL-10 that is recognized by commercially available antibodies. The transposons for these isolates were inserted into the genes for trehalose 6 phosphate, DnaK, and 23s rRNA for mil10.4, −13, and −14, respectively.

Given that mIL-10 is glycosylated,^30^ we wanted to ensure that it was bioactive to cells that would respond to IL-10 signaling. To address this, we isolated memory (CD44^hi^CD62L^low^) CD4^+^, CD8^+^, and γδ T cells from C57BL/6 mice. We non-specifically activated these cells by stimulating αβ T cells with plate-bound αCD3 and soluble αCD28 for 3 days. In the presence of supernatants from mIL-10-producing *C. mast* (mIL-10-*C. mast*), T cells proliferated to a lesser extent as compared to T cells stimulated in supernatants from wild-type (WT) *C. mast* (AS1) (Figure 2B). Similarly, the production of the proinflammatory cytokine, IFN-γ, was inhibited by supernatants from IL-10-*C. mast*, which supports the regulatory nature of mIL-10 during *in vitro* stimulations, demonstrating functionality (Figure 2C). Notably, the cytokine, IL-17, which is required for optimal protection of the ocular surface from spontaneous infection,^31^ was not inhibited by IL-10-*C. mast* supernatants (Figure 2D). Because IL-17 is not reduced in the presence of supernatants from IL-10-*C. mast*, we posited that IL-10-*C. mast* does not affect the host-microbe interactions concerning the ocular microbiome.

### IL-10-*C. mast* colonizes the mouse eye and stimulates host immune responses similar to WT *C. mast*

**Figure 3.**
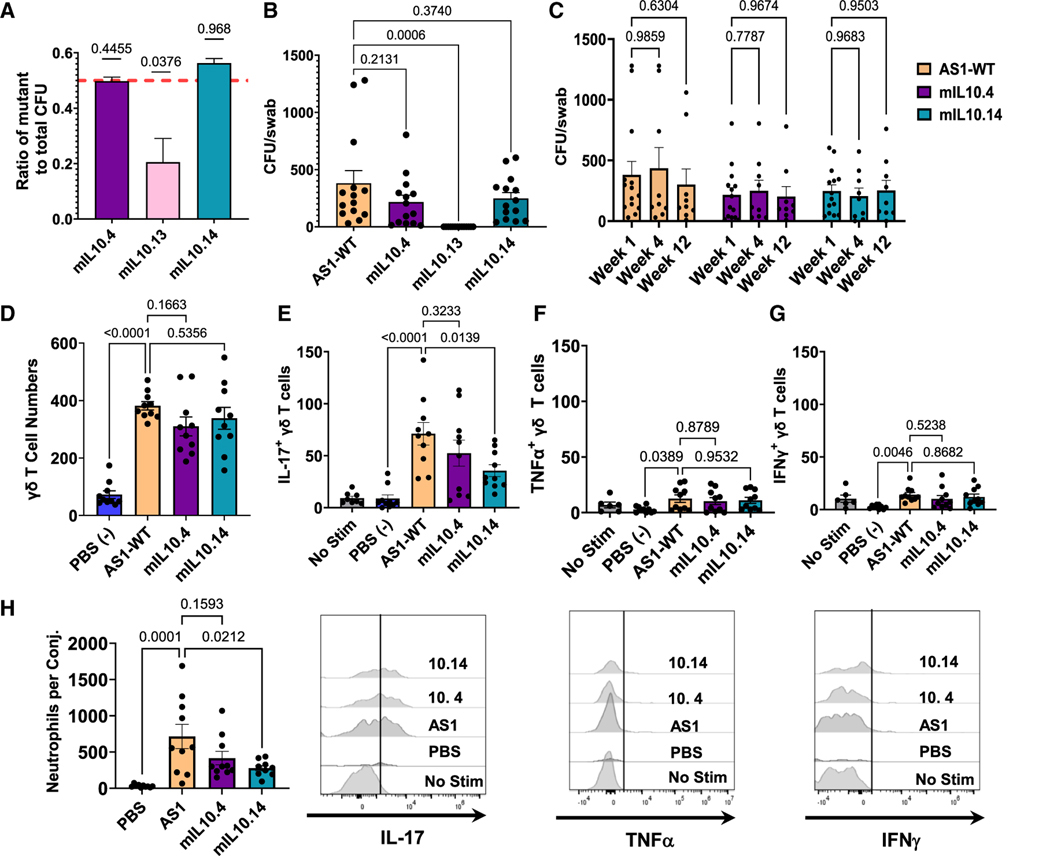
IL-10-*C. mast* colonizes mouse eyes and stimulates host immune responses similar to WT *C. mast* (A) Log-phase mutant and WT bacteria were equally mixed in antibiotic-free LB at 0.5 OD_600_. They were grown for 6 h before being plated on agar with and without antibiotics. Comparing the CFUs on each plate, the ratio of mutant to WT AS1 was determined. The bars represent the mean ratio of mutant CFUs to total CFUs ± SEM. Data were pooled from three independent experiments (*n* = 3), and *p* values were calculated using a one-tailed single-sample *t* test with an expected mean of 0.5. (B and C) 5 ×10^6^ CFUs of bacteria were applied to the ocular surface of C57BL/6 mice once every other day for a total of three inoculations. (B) One week after the final inoculation, the eyes of mice were swabbed, and the CFUs per swab were quantified using agar plates. (C) This was repeated at weeks 4 and 12 post-inoculum with the isolates that could colonize the eye. The bars represent the mean CFUs/eye ± SEM. Data were pooled from 3 experiments per time point, and the statistical *p* values were determined using (B) ANOVA or (C) type III ANOVA of fixed effects. (D–H) In two separate experiments, conjunctivae were collected from mice colonized for 2 weeks as previously described and processed for flow cytometry. (D–G) Cells were stimulated for 4 h with PMA/ionomycin in the presence of brefeldin A. After stimulation, cells were stained for flow cytometry, and γδ T cells were assessed for (D) total cell numbers, (E) IL-17 production, (F) TNF-α production, and (G) IFN-γ production. Flow plots represent typical staining patterns across samples. (H) In parallel, conjunctiva cells were stained for neutrophil markers (CD11b^+^Ly6C^−^ Ly6G^+^) and assessed by flow cytometry. (B–H) Symbols represent individual mice pooled from two independent experiments, and the bars represent the mean ± SEM number of cells. (D–H) Significance was determined by ANOVA.

To assess whether IL-10-*C. mast* would survive in a physiological environment, we performed *in vitro* and *in vivo* experiments to quantify fitness. First, mutant and WT strains of *C. mast* were grown to log phase and mixed at a 1:1 ratio. This allowed a direct comparison of growth between each mutant and WT *C. mast* (Figure 3A). After 6 h in liquid broth, isolates 4 and 14 did not have a significantly lower ratio of mutant bacteria than at the initial time point. Conversely, isolate 13 trended toward a reduced ability to compete with WT *C. mast,* showing a significantly reduced ratio of mutant vs. WT bacteria as in the initial culture. We next analyzed the mutants for their ability to colonize the mouse conjunctiva. Five million colony-forming units (CFUs) of log-phase mutant or WT *C. mast* were applied to the eye every other day for a total of 3 inoculations. After 1 week, the eyes were swabbed, and colonizing bacteria were quantified. Both isolates 4 and 14 were able to colonize the mouse eye with no significant change in CFUs compared to WT, while isolate 13 lacked the ability to remain at the ocular surface (Figure 3B). Notably, over a span of up to 12 weeks, both isolates 4 and 14 maintain their ability to remain at the ocular surface and are not eliminated by host immune responses (Figure 3C), and there were no statistical differences in CFU counts between WT *C. mast* and IL-10-*C. mast* at each time point. These data indicate that neither isolate 4 nor 14 was significantly less fit than WT *C. mast*; however, isolate 13 was significantly impaired in its ability to colonize the eye. Given that isolate 13 could not colonize the eye and that the transposon cassette was incorporated into the DnaK gene, we concluded that the transposon impaired the function of the critical DnaK chaperone protein and reduced the fitness of isolate 13 to the point that colonization was not possible.

To assess the therapeutic potential of IL-10-*C. mast*, we asked whether the secreted mIL-10 altered homeostasis at the ocular surface. First, we measured whether detectable levels of mIL-10 were produced within the conjunctiva of mice colonized with WT *C. mast*, IL-10-*C. mast*, or PBS. Even though detectable levels of mIL-10 were observed *in vitro*, we did not observe a significant increase in free mIL-10 within the conjunctiva of mIL-10-*C. mast*-colonized mice a week after inoculation (Table S1). To assess the immunity generated against mutant bacteria, we collected and processed conjunctivae from mice colonized for 2 weeks with bacteria and mice treated with PBS. Upon assessment of the conjunctival γδ T cell response, we found no differences in the number of γδ T cells (Figure 3D) and IL-17-producing γδ T cells (Figure 3E) after stimulation with PMA/ionomycin between AS1 and mIL10.4. We observed a slight but significant reduction in anti-*C. mast* immunity between AS1 and mIL10.14 in regard to IL-17 production from γδ T cells. Additionally, we found no appreciable differences in the production of TNF-α or IFN-γ after γδ T cell stimulation between AS1 and the transposon mutants; however, the presence of any of the bacteria correlated with a slight increase in cytokine-producing cells over the PBS-treated controls. Therefore, *C. mast* or the transposon mutants do not induce disparate cytokine responses at the ocular surface (Figures 3F and 3G). Downstream of γδ T cell IL-17, neutrophils were slightly reduced in the conjunctiva of mice colonized with IL-10-*C. mast* (Figure 3H); however, we concluded from the sum of these data that mIL-10-*C. mast* does not dramatically alter the immune landscape within the conjunctiva compared to the conjunctiva of mice colonized with the WT strain of *C. mast.*

### IL-10 produced by *C. mast* can enhance wound healing in an IL-10-dependent manner

**Figure 4.**
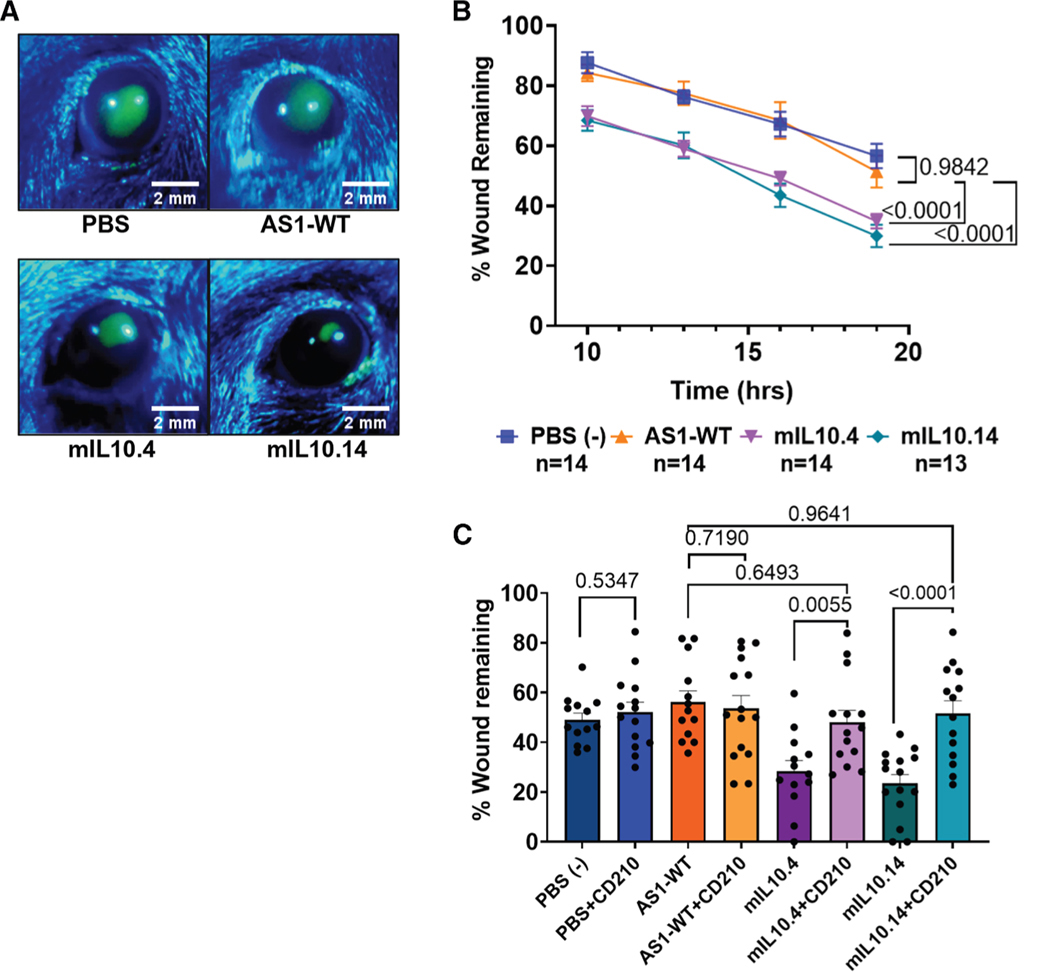
IL-10 produced by *C. mast* can enhance wound healing in an IL-10-dependent manner Mouse eyes were inoculated with 5 × 10^6^ CFUs of mutant or WT bacteria once every other day for three inoculations. One week after the final inoculation, a 2.5 mm abrasion of the corneal epithelium was made using an Alger brush. Fluorescein was used at the time of injury, then at 10 h and every 3 h after to measure the area of unhealed epithelium of each mouse. (A) Representative images of unhealed area of corneas 19 h after wounding. (B) Percentage of wound remaining was determined by measuring areas of corneal wounds using FIJI at each time point, and this area was compared to the T0 values. The bars represent the mean percentage of wound remaining ± SEM. Data are pooled from three independent experiments. The area under each curve was calculated, and the mean and SD were evaluated for significance using an ANOVA. (C) Twenty-four hours prior to corneal wounding, mice were given an intraperitoneal (i.p.) injection of αCD210 to neutralize the IL-10R. The percentage of wound remaining was calculated at T = 19 h. Each dot represents a single mouse, and the bars represent the mean percentage of wound remaining ± SEM. Data were pooled from three independent experiments, and statistical significance across all samples was determined using an ANOVA. Differences between samples treated and untreated with CD210 were analyzed using an unpaired *t* test.

We used a mouse model of corneal wound healing to assess the physiological effects of IL-10-*C. mast* at the ocular surface due to the advantageous nature that IL-10 has on the healing of corneal wounds. One week after mice were ocularly colonized with WT *C. mast,* IL-10-*C. mast*, or PBS, equal-size (2.5 mm) abrasions of the corneal epithelium were made using an Alger brush. Fluorescein imaging was used to assess the size of the wounded area at the time of injury and until 19 h after injury (Figures 4A and 4B). IL-10-secreting bacteria significantly increased the rate of wound healing compared to controls. To ensure that the observed effects were directly related to IL-10 signaling, we administered IL-10R blocking antibody (CD210) intraperitoneally 24 h prior to wounding, and we observed fluorescein staining at t = 0 and t = 19 h (Figure 4C). Our results showed that wound healing was significantly slowed after CD210 administration in mice colonized with IL-10-*C. mast.* CD210-treated mice that were colonized with IL-10-*C. mast* had the same rate of wound healing as PBS and WT AS1 controls did, supporting the notion that engineered IL-10 was biologically functional and was responsible for the increased rate of wound healing after corneal injury. Together, these findings outline the therapeutic potential of engineering an ocular resident microbe to deliver bioactive therapeutics directly to the ocular surface to treat disease.

### hIL-10 produced by *C. mast* is functional when colonized on the mouse eye

**Figure 5.**
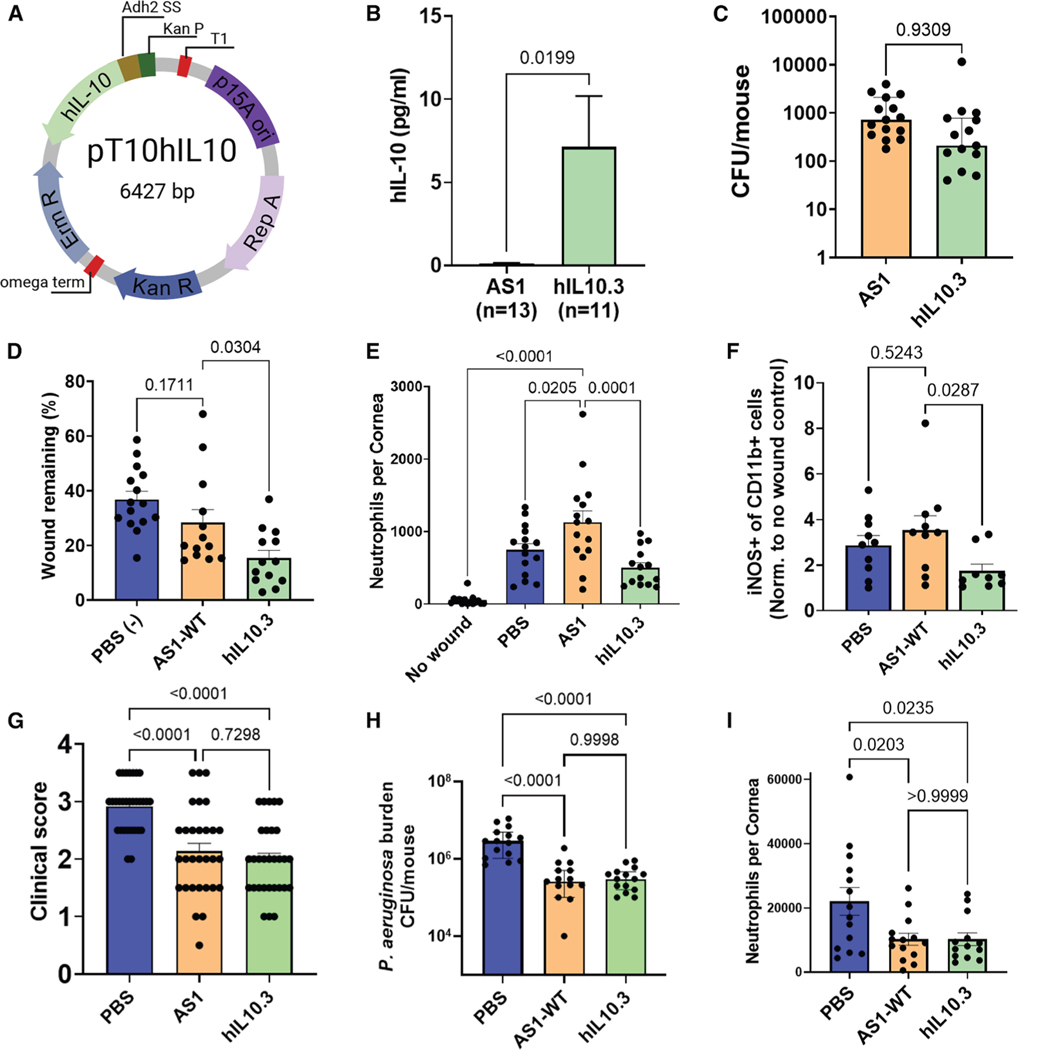
Human IL-10 produced by *C. mast* is functional when colonized on the mouse eye (A) The human IL-10 gene was fused to the native *C. mast* secretion signal and expressed on plasmid pT10hIL10. (B) After transformation into *C. mast*, the supernatant of positive transformants was subjected to hIL-10 ELISA and compared to WT AS1 using a Student’s *t* test. (C–I) Mouse eyes were inoculated with 5 × 10^6^ CFUs of hIL-10-expressing or WT bacteria once every other day for three inoculations. (C and D) Two weeks after the final inoculation, mouse eyes were swabbed and plated to count colonization. Significance was evaluated using a Student’s *t* test. (D and E) After colonization was verified, a 2.5 mm abrasion of the corneal epithelium was made using an Alger brush. (D) Fluorescein was used at the time of injury and then at 19 h to measure the area of unhealed epithelium of each mouse. The percentage of wound remaining was measured using FIJI. The bars represent the mean percentage of wound remaining ± SEM. Data were pooled from three independent experiments, and statistical significance across all samples was determined using an ANOVA. (E and F) Mouse corneas were collected 10 h after wounding and analyzed via flow cytometry. (E) Neutrophils were quantified by using the Ly6G^+^Ly6C^int^ population of CD11b^+^ cells. (F) iNOS expression was assessed by flow cytometry on CD11b^+^ cells in the cornea. (G–J) Two weeks after inoculation, *C. mast* colonization was verified, and then the mouse eyes were scratched before 10^5^ CFUs of *P. aeruginosa* were added to each eye. 48 h after infecting the eyes, (G) keratitis scores were measured, (H) one whole eye was homogenized and plated to evaluate *Pseudomonas* burden, and (I) the cornea of the remaining eye was processed for neutrophil quantification by flow cytometry. (C–F, H, and I) Symbols represent individual mice or (G) individual eyes of mice. Data are pooled from at least three independent experiments. Two representative experiments are displayed in (F). (B–I) Error bars represent the standard error of the mean.

In order to advance this technology for patient treatment, a human version of the IL-10 gene needed to be expressed by our LBP. We engineered a plasmid with both kanamycin and erythromycin resistance genes to secrete the product of a harmonized version of the hIL-10 gene using the primers listed in Table 1 (Figure 5A). This plasmid was transformed into WT-AS1 *C. mast*, and supernatant from positive transformants was analyzed for hIL-10 via ELISA (Figure 5B). The transformant that secreted the most hIL-10, hIL10.3, was used to inoculate mice in parallel with mice inoculated with WT *C. mast*. After 2 weeks, colonization was analyzed, and no significant difference in the number of CFUs was found between WT and hIl10.3 (Figure 5C). Mice colonized for 2 weeks with WT or hIL10.3 or PBS control mice were used in a wound healing experiment as described above. Nineteen hours after wounding, the mice colonized with the hIL-10 secreting *C. mast* had significantly less wound remaining than AS1 or the PBS control (Figure 5D). The conjunctivae of mice colonized and wounded similarly were also analyzed via flow cytometry. Ten hours after wounding, corneas were collected and processed. The corneas of mice colonized with hIL10.3 had significantly fewer neutrophils and inducible nitric oxide synthase (iNOS)-expressing CD11b^+^ cells (Figures 5E and 5F).

To identify if hIL-10-*C. mast* compromised the ability of the ocular surface to resist infection and pathology, we challenged the corneas of colonized mice with *Pseudomonas aeruginosa*, similar to our previous studies.^26^ We observed that WT *C. mast* and hIL-10-*C. mast* significantly enhanced the ability of the cornea to resist infection with *P. aeruginosa*. Specifically, both AS1 and hIL10.3 were associated with lower clinical scores (Figure 5G). Lower clinical scores were associated with a lower *P. aeruginosa* burden and neutrophil infiltration in AS1- and hIL10.3-inoculated eyes (Figures 5H and 5I). We concluded from these data that hIL-10 delivered via the eye-colonizing bacteria *C. mast* aids in wound healing without compromising the inherent immune responses that protect the eye from pathogenic infection.

### hIL-10 produced by *C. mast* is functionally active in cultured human cells

**Figure 6.**
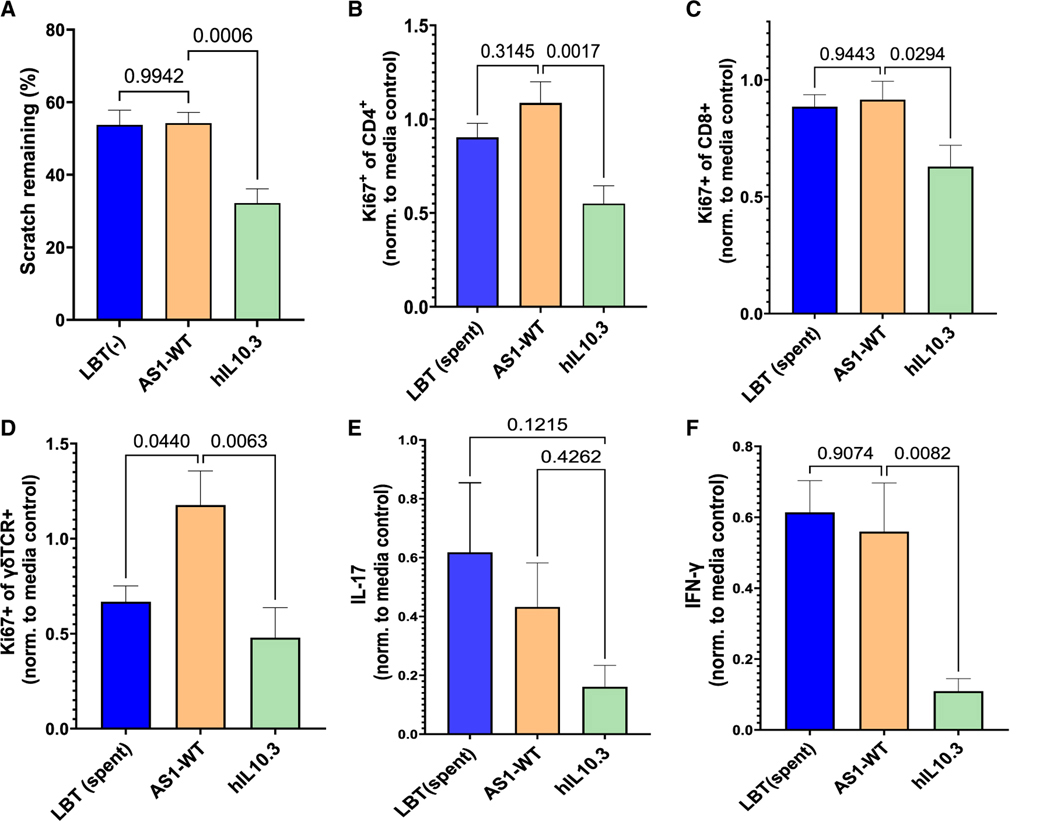
Human IL-10 produced by *C. mast* is functionally active in cultured human cells (A) Human corneal limbal epithelial cells were grown in a monolayer on a flat-bottom 48-well tissue culture plate. Scratches were made using a p200 pipette tip, and supernatants from each culture were added in triplicate. Scratches were analyzed using FIJI at t = 0 and t = 8 h. This was repeated 3 times, and 3 technical duplicates were averaged for each (*n* = 9). (B–D) Human PBMCs (*n* = 7) were stimulated with Dynabeads, and media or culture supernatants were added in triplicate to each well. After 72 h, the supernatant was removed, and the cells were subjected to flow cytometry to measure the proliferation of each culture. (E and F) The supernatant from PBMC culture was used in ELISA to determine IL-17 and IFN-γ production. (B–F) Error bars represent the standard error of the mean.

We next wanted to assess how hIL-10 produced by *C. mast* influenced human cells *in vitro*. For this, we performed *in vitro* scratch assays with human corneal limbal epithelial cells (HCLEs),^32^ where HCLEs are grown to a monolayer in a culture dish and a pipette tip is used to create a wound through the center of the culture dish. Similar to our *in vivo* mouse studies, supernatants from hIL-10-secreting bacteria sped wound healing of human cells *in vitro* (Figure 6A). In addition, we acquired peripheral blood mononuclear cells (PBMCs) from healthy human subjects, and we stimulated these cells non-specifically with αCD3 and αCD28 beads. Notably, we observed significant reductions in the proliferation of CD4, CD8, and γδ T cells *in vitro* (Figures 6B–6D). Additionally, IFN-γ, but not IL-17, produced in these cultures was also significantly reduced, suggesting that the supernatant of hIL-10-*C. mast* is sufficient to limit the inflammatory potential of human immune cells without necessarily compromising anti-commensal immunity. From these data, we concluded that IL-10-*C. mast* has the potential to limit excessive inflammatory responses in human cells and may be a viable strategy to treat ocular surface inflammation.

## DISCUSSION

The ocular surface continues to be a difficult tissue for the application of therapeutics, given the sensitive nature of the cornea, the aversion of patients to touching the eye, and the constant flow of tears across the ocular surface. Current standards of care for local delivery of therapeutics include invasive and/or laborious methods such as subconjunctival injections and topical application up to once every 2 h. To remedy this, we devised a strategy where we engineered the known eye colonizer *C. mast* to produce and secrete an anti-inflammatory and pro-wound-healing cytokine. In this study, given the anti-inflammatory and pro-healing qualities of IL-10, we decided to engineer *C. mast* to secrete IL-10 at the ocular surface.

Despite *Corynebacterium* spp. having been engineered in the past, most studies have focused on engineering *C. glutamicum*—a soil-habiting bacterium—to produce amino acids in an industrial production setting^33^; our study, however, focused on genetically manipulating *C. mast* for the purposes of generating a therapeutic delivery vehicle or an LBP. The benefits of LBPs are that colonization with them negates the need for repeated treatment regimens that reduce compliance. Similarly, LBP therapies could theoretically reduce production costs, as the therapeutic is derived from the bacterium rather than synthetic methods. Furthermore, LBPs have proven effective in pre-clinical and some clinical trials involving Crohn’s disease and irritable bowel syndrome (IBS).^20,22^ More recently, IL-10 produced in *Mycoplasma pneumoniae* was shown to be effective at limiting the inflammatory response related to *P. aeruginosa* lung infection.^34^ With that, we hypothesized that IL-10 delivered by an eye-colonizing microbe may be beneficial for ocular surface health. Indeed, the production and release of IL-10 from *C. mast* resulted in more efficient tissue repair at the ocular surface after corneal injury. This suggests that this may be an effective method of treatment for traumatic ocular surface injuries, which affect over 1 million Americans per year.^1^ Furthermore, refinement of this technology to include other relevant proteins or naturally derived factors may prove useful in other models of ocular surface disease, eliminating laborious treatment strategies that are associated with the topical application of eye drops.

This strain of *C. mast* was originally isolated from the ocular surface and skin of mice, but unlike laboratory strains of bacteria, genetic tools and protocols to manipulate *C. mast* have not been well developed. Recently, our group used transposon mutagenesis to incorporate the gene of mCherry into the *C. mast* genome,^27^ and we used this same system to incorporate mIL-10 into *C. mast*. Specifically, we needed to identify a signal sequence that would direct *C. mast* to secrete the factor of interest rather than relying on lysing bacteria as in previous studies.^35^ For this, we generated a PhoZ transposon library to discover a strong Sec/SPI signal peptide, which is a standard signal that is trafficked outside the cell by the Sec translocon and cleaved by the SPI. Mutants that could successfully deliver the PhoZ outside the cell turned blue when the BCIP was added extracellularly. This provided us with a high-throughput and effective tool to identify the signal sequence that would work most ideally for *C. mast.* While mCherry and PhoZ were relatively easy to express in *C. mast*, mIL-10 with an attached signal sequence proved more difficult, as we were able to identify fewer transposon mutants. This trend continued when trying to express hIL-10. All transposon mutants collected when trying to express hIL-10 from the genome of *C. mast* were overstressed and grew poorly. Because of this, we elected to express hIL-10 from a plasmid, which seemed to allow the bacteria to thrive and be able to survive in a clinical environment.

A difficulty in modifying host immune responses is the balance of inflammation regulation, wound repair, and infection prevention. Given that we induced expression of IL-10, an immune regulatory cytokine, we were concerned that the continual production and release of IL-10 may affect the growth and control of *C. mast*, resulting in *C. mast* becoming a pathobiont. Notably, we observed that mIL-10 from *C. mast* reduced the inflammatory cytokine IFN-γ, but it did not affect the production of IL-17 from γδ T cells *in vitro*, which is produced in response to *C. mast* colonization. More importantly, we monitored mice colonized with WT *C. mast* and IL-10-*C. mast* for multiple months and found that neither caused clinical signs of pathology during colonization. Even though transgenic bacteria remained at the ocular surface long term, the *in vivo* production of IL-10 at the ocular surface remained below the limit of detection for commercial reagents (Table S1). Furthermore, without wounding, the mIL-10-*C. mast* did not significantly affect the number of γδ T cells or cytokines produced by them in the conjunctiva; however, the number of neutrophils recruited did significantly decrease compared to the WT control. Neutrophils were also reduced in wounded corneas that were colonized with hIL-10-*C. mast.* Both h- and mIL-10-producing bacteria sped wound healing, and an antibody blockade of IL-10 signaling abrogated the beneficial effects of mIL-10-*C. mast*, showing that the increased rate of healing was directly related to the addition of the IL-10-producing bacteria. From this, we conclude that the host immune system is likely more sensitive to IL-10 *in vivo* than commercially available reagents. Furthermore, given the delicate balance between regulating inflammation and immune suppression, we conclude that the low level of *in vivo* IL-10 production from IL-10-*C. mast* is advantageous in this system because the bacterium is less likely to affect the control of infectious agents. This is supported by our data showing that *P. aeruginosa* clearance is not compromised when the conjunctiva is colonized by IL-10-producing bacteria. Similarly, there were no differences in the host response, IL-17 from γδ T cells, to colonization with either WT *C. mast* or IL-10-*C. mast*. Therefore, we concluded that even though IL-10-*C. mast* produced an immune-regulating cytokine, it is unlikely that this genetically engineered microbe would become pathogenic in an immunocompetent host. Because of our engineered *C. mast*’s ability to remain long term, induce IL-17 immunity similar to WT, and lack of pathology, we demonstrate that use of engineered *C. mast* as an LBP has the potential to be well tolerated and beneficial in a clinical setting.

Most LBP research has focused on influencing the host-gut microbiome axis; however, more attention is being directed at low-biomass sites. Recently, LBPs have proven useful in the lungs^34,36^; however, here we have demonstrated proof of concept of an LBP that can colonize the ocular surface. Specifically, the purpose of this LBP is to treat ocular surface wounds, but this technology can likely extend to other diseases at the ocular surface. Here, we simplified the system to ensure that we reduced as many variables as possible. Indeed, we were able to focus our efforts on identifying a secretion signal and ensuring that functional IL-10 was produced during colonization. Future iterations of this technology may rely on other cytokines and/or factors that may better tune the ocular surface microenvironment to encourage homeostasis. Specifically, there may be proteins or cytokines that help reduce inflammation related to Sjogren’s syndrome and severe dry eye disease. Alternatively, it may be possible to use this technology to produce growth factors that may enhance the longevity of adoptively transferred stem cells or reduce the possibility of corneal graft rejection. This LBP secreted physiological levels of functional IL-10 in culture and *in vivo* at the ocular surface, which demonstrates that this technology may negate the need for burdensome treatment regimens that need to be applied to the eye multiple times a day; however, more work is required prior to implementing this technology in the clinic. Overall, the well-tolerated nature of this bacterium and its efficacy in delivering cytokines to the ocular surface highlight the possibilities of using engineered ocular-resident microbes as an alternative therapy for treating ocular surface diseases.

### Limitations of the study

Despite the *in vivo* neutralization of IL-10 using systemic injections of the αIL-10R, we could not achieve detectable levels of IL-10 in the tears after colonization. These data highlight that this technology is not yet tunable in that we cannot modulate the rate of secretion of a factor of interest. This may be due to a number of reasons but could include the bacterial modulation of promoter activity in liquid broth and at the ocular surface. The inhospitable nature of the ocular surface is well-documented, and this may force the bacteria into a stress response, which can alter the activity of various promoters *in vivo*. In future studies, identification and use of conditional promoters that are more or less responsive to environmental factors at the ocular surface may allow for adjustments in the amount of effectors of interest delivered to the eye throughout the duration of the therapy.

The pleiotropic effects of IL-10 on host physiology make it difficult to confirm exactly how this therapy functions *in vivo*. That being said, we have shown that the supernatants from this bacterium can limit immune responses related to cytokine production and cell proliferation. Additionally, we showed that supernatants from this bacterium can speed the closure of a wound *in vitro* without exogenous immune cells, suggesting that IL-10 could also be functioning on epithelial cells to speed proliferation. While our *in vivo* studies cannot explicitly identify whether IL-10 is functioning exclusively on one population of cells, the abrogation of the effect using αIL-10R confirms that IL-10 is functional *in vivo*. Together, we concluded that IL-10 is likely functioning in multiple populations of cells. Future studies are planned to assess the phosphorylation of STAT3 in specific populations of cells, which is directly downstream of IL-10 signaling.

Finally, as with any study investigating the manipulation of microbiomes, there are concerns about peripheral or unforeseen effects. Even though we observed no histopathological effects of this microbe, we did not account for times of immune compromise, tolerance, or suppression. Our study was limited to 12 weeks of colonization; it is possible that long-term colonization may eventually contribute to immune suppression that could lead to dysbiosis, outgrowth of genetically engineered bacteria, or other, more severe diseases. These possibilities highlight the necessity of developing a way to control this bacterium *in vivo*. Specifically, incorporating genetic switches or specific antibiotics that allow selective removal of the engineered bacterium may limit the possibility of global immune suppression.

Overall, there are likely to be more iterations of this technology before it can be realistically implemented in a clinical trial; however, this proof of concept opens the possibility of harnessing the ocular microbiome to treat ocular diseases.

### RESOURCE AVAILABILITY

#### Lead contact

Requests for further information and resources should be directed to and will be fulfilled by the lead contact, Anthony St. Leger (anthony.stleger@pitt.edu).

#### Materials availability

There are restrictions on the availability of the hIL-10 plasmid because it is patent pending. Bacterial strains generated in this study will be made available upon request, but we may require payment and/or a completed materials transfer agreement if there is potential for commercial application. All unique/stable reagents generated in this study are available from the lead contact with a completed materials transfer agreement.

#### Data and code availability

- The mIL-10 transposon sequences are available in the GenBank data-base (GenBank: PP471979.1 & GenBank: PP471980.1).
- The hIL-10 plasmid sequences are patent pending.
- The datasets supporting the conclusions of this article have been deposited at Mendeley Data: https://doi.org/10.17632/kwhys2zfwd.1.
- Microscopy data reported in this paper will be shared by the lead contact upon request.
- This paper does not report original code.
- Any additional information required to reanalyze the data reported in this paper is available from the lead contact upon request.

## STAR★METHODS

### EXPERIMENTAL MODEL AND STUDY PARTICIPANT DETAILS

#### Bacterial culture and genetic constructs

*C. mastitidis AS1*^26^ was isolated from ocular swabs and cultured with LB media containing 0.5% Tween-80 grown aerobically at 37°C. For T cell culture experiments cell culture media was used consisting of DMEM with 10% FBS; 1% NEAA, glutamax, HEPES, and sodium pyruvate. Bacteria added to T cell culture used the same cell culture media with the addition 0.5% corn oil and 0.05% w/v casamino acids. *P. aeruginosa* strain PAC [PMID: 11581056] is a fluoroquinolone resistant and invasive keratitis isolate used in this study. PAC was stored at −80°C, streaked on trypticase soya agar supplemented with sheep erythrocytes (5%), and grown in lysogeny broth (LB). Bacteria density was measured by optical density at 600 nm and adjusted to 10^^^5 CFU in 5 μL.

The phosphatase Z gene was isolated via PCR from DNA extracted from *Enterococcus faecalis* using primers indicated in Table 1. The *aphA-3* kanamycin resistance gene was isolated from a previously created plasmid, pTony3,^27^ and the erythromycin resistance gene cassette was created by using the same promoter attached to a codon harmonized erythromycin resistance gene of *C. striatum*. Codon harmonized mIL-10 and hIL-10 were ordered as gBlock Gene Fragments (IDT, USA). Codon harmonization was performed using the online Galaxy software, Codon Harmonizer.^38^ The hIL-10 secreting plasmid was created using the *E. coli* p15a origin of replication and kanamycin resistance genes from plasmid pTony3^25^ with a native corynebacteria origin of replication and the harmonized hIL-10, erythromycin resistance cassette. All newly created sequences can be found in the GenBank database (GenBank: PP471979.1 & GenBank: PP471980.1) and all primers used are detailed in Table 1. Patent is pending for the harmonized hIL-10 plasmid construct.

#### Mice

Female C57BL/6 mice purchased from Jackson Laboratories (Bar Harbor, ME, USA) were housed in the Animal Resource Facility at the University of Pittsburgh Medical Center (Pittsburgh, PA, USA) and colonized with *C. mast* at 6 weeks of age in all experiments according to our previous inoculation schemes. The use of animals was in accordance with the ARVO Statement for the Use of Animals in Ophthalmic and Vision Research. University of Pittsburgh IACUC approval number: 23063056.

### METHOD DETAILS

#### Generation of the mIL-10 secretion cassette

Secretion signals for *C. mast* were identified by creating a phosphatase (PhoZ) transposon library. Using Overlap extension PCR (OEP) and the primers indicated in Table 1, promoterless *phoZ* lacking its secretion signal from *Enterococcus faecalis* was fused with an *aphA-3* kanamycin resistance gene from a previously created plasmid, pTony3.^27^ This was the transposome that was inserted randomly into the wild-type *C.mast* genome using EZ-Tn5(Lucigen), as previously described.^27^ Positive mutants secreting PhoZ were selected for on kanamycin plates (100 μg/mL) supplemented with BCIP (5-bromo-4-chloro-3^′^-indolyphosphate p-toluidine salt) (25μg/ml) that turns colonies blue when phosphatase is secreted. Blue colonies were grown in broth to log phase, and supernatant was collected and analyzed using the 1-Step PNPP Substrate Solution from ThermoFisher and repeated in triplicate. The genomes from the best secreting isolates were sequenced via Illumina whole genome sequencing,150 Mbp: 66 times genome coverage, to find the insertion site of the transposome cassette. The insertion site sequence was analyzed using signalP 5.0^29^ to find the strongest predicted secretion signal.

#### mIL-10 production and functionality *in vitro*

The DNA sequence of the strongest signal was added to a codon harmonized mouse-IL-10 gene preceded by a strong constitutive promoter in a synthesized double stranded DNA fragment (gBlock Gene Fragment, IDT, USA). This was fused with a harmonized erythromycin resistance gene from *C. striatum* using overlap extension PCR to create the new transposome cassette and inserted into the *C.mast* genome. Positive isolates were screened using antibiotic selection (100 μg/mL) with over 200 positive mutants collected. These were grown to log phase and screened for mIL-10 production in their supernatant using IL-10 Mouse Uncoated ELISA kit (ThermoFisher) and normalized to OD_600_. In three separate experiments, the best cytokine secreting isolates were verified by growing each to exactly 0.5 OD_600_ and quantifying supernatant mIL-10. The functionality of the cytokine was tested *in vitro* by analyzing the effect of mutant supernatant on T cell proliferation. Naive (CD44^−^ CD62L^+^) or memory (CD44^hi^CD62L^low^) CD8^+^, CD4^+^ and γδT-cells were isolated by fluorescence-activated cell sorting (FACS) on a Sony Biotechnology MA900 cell sorter. After sorting, cells were labeled with a proliferation dye (Cell Trace Invitrogen) and stimulated for 3 days with plate-bound αCD3 (1mg/ml) and soluble αCD28 (5mg/ml). Six hours after the initial stimulation, 10 μL of supernatant from log-phase bacterial cell culture broth of AS1 or mIL-10 secreting mutants. After stimulation, proliferation was measured via flow cytometry using a Beckman Coulter Cytoflex LX. T cell culture supernatants from these assays were then assessed for IFN-γ and IL-17 production using ELISA (BioLegend). Antibody panels used are detailed in Table S2.

#### Fitness of mIL-10 transposon mutants

To assess the fitness of the mIL-10 secreting mutants they competed with *WT C. mast* in the same broth culture through three separate experiments. Transposon mutants and WT AS1 were grown to log phase in liquid media, pelleted, washed and resuspended at 0.5 OD_600_ in media without antibiotic. One mL from each transposon mutant resuspension was mixed with 1 mL of WT and allowed to grow together for 6 h. The cultures were then 10-fold serially diluted to 10^−8^ and 100mL was plated on agar both with and without antibiotic to select for the mutant. Comparing the CFU on each of the plates allows for the quantification of the ratio of mutant bacteria contained in the culture.

These isolates were also evaluated *in-vivo* for their ability to colonize the mouse eye. Transposon mutants and WT AS1 were grown to log phase in liquid media Then pelleted and resuspended at 1×10^6^ CFU/ml in PBS. In three separate experiments, five mice per sample were inoculated with 5 μL of each mutant, control WT and PBS dropped onto mouse eyes that had been brushed with a sterile swab to disrupt the tear film. This was repeated every other day for a total of 3 inoculations. One week after the last inoculation mouse eyes were swabbed and plated on selective media. Colonies were counted and compared to the colonization rate of WT AS1 to determine *in vivo* fitness. This was repeated on mice colonized with WT and mutant *C. mast* for 4 weeks and 12 weeks to compare retention and survival of the mutant strains.

#### In-vivo immunologic response to transposon mutants

Mice were inoculated as described previously; two weeks after the final inoculation mice were sacrificed and the conjunctiva was excised. The tissue was minced with scissors and broken down with collagenase before being passed through a 45μm filter and washed. The cells were then passed through a falcon filter capped tube (catalog# 08–771-23) and resuspended in cell culture media. Half of the sample was stimulated for 4 h at 37°C using a leukocyte activation cocktail (BD Pharmingen Leukocyte Activation Cocktail, with BD GolgiPlug) the other half was used to stain for neutrophils and iNOS expressing CD11b^+^ cells using flow cytometry. Antibody panels used are detailed in Table S2. Samples were analyzed on a Beckman Coulter Cytoflex LX or a Sony ID7000 spectral cytometer.

#### mIL-10 effect on *in-vivo* corneal wounds

To evaluate the effect of the mIL-10 producing mutants on the ocular surface we inoculated the eyes as previously described and bacteria were quantified to ensure colonization and no contamination. Two weeks after the last inoculation a 2.5mm epithelial corneal wound was made on the right eye of each mouse using an Alger brush without disrupting the basement membrane. A 0.01% Fluorescein solution was then dropped onto the eye and pictures were taken at T = 0. Fluorescein-stained cornea pictures were again taken at T = 10 h and every three hours after until the first wounds showed full closure at 22 h. The size of the wound at each time point without full closure was measured by increasing the saturation of only the green pigment in the image and using the analyze particles function of Fiji (ImageJ). The size at each timepoint was then compared to the size at T = 0 to find the amount of healing at each time point for each individual mouse. These data were replicated with three total experiments.

Finally, the mIL-10R was blocked *in vivo* using CD210 antibody administration (Bio X Cell). Two weeks after the final bacterial inoculation, 200 mg of CD210 in 1X PBS was injected intraperitoneally (IP). Controls were given IP injections of IgG. Twenty-four hours after injection corneal wounds were performed and analyzed at t = 0 and t = 19 h as previously described. The amount of healing for each isolate was compared between CD210 treated mice and IgG injected mice as well as each isolate compared to the WT-control.

#### Creation human IL-10 secreting live biotherapeutic

First, transposon mutagenesis was used as described previously to create a human IL-10 secreting strain; however, all positive transposon mutants proved extremely unfit and were not suitable for analysis. Therefore, we shifted to a plasmid expression system. Using the *E. coli* origin of replication and kanamycin resistance genes from pTony3^27^ we added a corynebacterium specific origin of replication, the erythromycin resistance gene used in the mIL-10 cassette and the hIL-10 secretion construct. The secretion signal from the mIL-10 cassette along with a verified constitutive promotor was used to create the hIL-10 secretion construct. This plasmid, named pT10hIL10, was first transformed into *E. coli* top10 (Invitrogen) competent cells for replication before being extracted and transformed into *C. mast AS1* competent cells and screened on fosfomycin (100μg/mL) and kanamycin (50μg/mL) LBT selection plates. Positive transformants were verified via PCR and growth in both kanamycin and erythromycin (100μg/mL) containing broth. Human IL-10 secretion was verified using IL-10 Human Uncoated ELISA kit (ThermoFisher) performed in the same way as described for the mIL-10 isolates.

#### hIL-10 functionality on mouse cornea

The hIL10 expressing *C. mast* was also evaluated for its ability to colonize and affect the ocular surface of the mouse eye. In 3 separate experiments, mouse eyes were inoculated as described previously with PBS, *WT C. mast,* and hIL10.3; after 2 weeks mouse eyes were swabbed and bacteria plated on LBT plates containing appropriate antibiotic. After colonization was confirmed, corneal wounds were performed on mice as described previously. Wound measurement was analyzed at t = 0 and t = 19 h and compared across samples. Furthermore, in 2 additional experiments, mice were colonized and wounded as above and 10 h after wounding corneas were excised and processed for flow cytometry to assess neutrophil numbers and iNOS expression in CD11b+ cells, similar to the conjunctiva processing described previously. Antibody panels used are detailed in Table S2.

The ability of the hIL-10 *C. mast* to protect the eye from pathogenic infection in the same way that *WT C. mast* does was then tested. Mice were inoculated with hIL-10, *WT C. mast* or PBS as previously described. Two weeks after inoculation, eyes were scratched and 100,000 CFU of *P. aeruginosa* was added to each eye. 48 h after infection the eyes were evaluated for clinical scores of microbial keratitis^39^ before eyes were extracted for analysis. One eye from each mouse was homogenized, diluted 10^−5^ and plated on LB to count the *P. aeruginosa* burden. The cornea from the other eye was processed for flow cytometry according to the corneal wounding experiment above. Antibody panels used are detailed in Table S2.

#### hIL-10 functionality on human cells

First, we evaluated the effect that the hIL-10 producing biotherapeutic had on Human Corneal Limbal Epithelial cells (HCLEs) during a scratch assay in three separate experiments. HCLEs were grown in Keratinocyte-SFM media (gibco) with Bovine Pituitary Extract, 0.2ng/mL Epidermal Growth Factor and antibiotics to 90% confluency. They were then collected and 60,000 cells were plated per well in a 48 well plate. and let to grow for 24 h to confluency. At the same time wild-type AS1 and hIL10 cultures were inoculated and grown to log phase. In the HCLE culture plates a straight line was scratched on each well using a p200 pipette tip held at a 90° angle then washed with 200μL room temperature media once to remove dead and scraped off cells before adding another 200μL for growth. Each well was imaged immediately afterward and 10μL of bacterial supernatant was added from three separate cultures in duplicate. LBT was used in triplicate as a control. After 8 h each well was imaged again and the difference in size of the scratch between t = 0 and t = 8 was analyzed using ImageJ.

Next the ability of the hIL-10 biotherapeutic to modify the immune response of human t-cells was evaluated by analyzing the effect they had on stimulated PBMCs using flow cytometry. First frozen PBMCs were acquired from StemCell from 7 individuals over the course of 3 separate experiments. PBMCs were plated at 100,000 cells per well were plated in a tissue culture treated 96 well plate and stimulated using Dynabeads Human T-Activator CD3/CD28. Wild-type and hIL-10 secreting C. mast were grown to log phase in LBT and diluted to 0.5 OD_600_. Bacterial cultures were pelleted and 10μL of supernatant was added to the stimulated PBMCs as well as a protein free spent LBT control and left to grow for 3 days. Spent LBT was made from supernatant of the AS1 culture in each experiment; 1mL of supernatant was filtered through a 0.2μm filter then heated to 100°C for 30 min. After heating the supernatant was spun at 13K Xg for 10 min to pellet the denatured proteins. The supernatant from the pelleted proteins was filtered though a 5KD protein filter to collect all any additional proteins that may have been left. After stimulation, proliferation was measured via flow cytometry using a Beckman Coulter Cytoflex LX. Antibody panels used are detailed in Table S2. T cell culture supernatants from these assays were then assessed for IFN-γ and IL-17 production using ELISA (BioLegend).

### QUANTIFICATION AND STATISTICAL ANALYSIS

All ANOVA and Student’s t-tests were performed using Graphpad PRISM software. Comparisons across more than 2 samples used an ANOVA with Dunnett’s multiple comparisons test for all individual differences. Figure 2A uses an RM one-way anova to account for variation across experiments. When only 2 samples were compared a Student’s *t* test was performed. For the *in vitro* fitness comparisons (Figure 3A) a one-tailed single sample *t* test was performed with an expected mean of 0.5. For colonization over time with mIL-10-*C. mast* a mixed effects analysis was performed. When comparing corneal epithelial wounds with and without IL-10R blocking an unpaired Student’s *t* test was used. Only wounds that healed at least 15% after 19 h were included in the analysis. All error bars represent standard error of the mean.

## Supplementary Material

1

SUPPLEMENTAL INFORMATION

Supplemental information can be found online at https://doi.org/10.1016/j.celrep.2026.117064.

## Figures and Tables

**Table 1.** Primers used in this study

Primer name	Sequence	Use

PhoZ NcoI F	5^′^tacactCCATGGactgTTAATTAAatctgaacagaaaagc3^′^	amplification of PhoZ from *E. feacalis*
PhoZ NotI R	5^′^tacactGCGGCCGCcgatGACGTCttatttaccaat3^′^	amplification of PhoZ from *E. feacalis*
Kanp Nco F	5^′^ATCTCCATGGtaagataatatatcttttatatagaagatatcgc3^′^	amplification of Kanamycin resistance gene
Kanp Mfe R	5^′^ccacCAATTGattTCCTTCCTCTTttc3^′^	amplification of Kanamycin resistance gene
PhoZ Kan Homo R	5^′^cataaagcttgctcaatcaatcaccGACGTC ttatttaccaatacctttatctttaatc3^′^	homologous overlap of Kanamycin resistance gene cassette for OEP
Kan PhoZ homo F	5^′^gattaaagataaaggtattggtaaataaGACGTC ggtgattgattgagcaagctttatg3^′^	homologous overlap of PhoZ gene cassette for OEP
PhoZ TN5 F	5^′^P’CTGTCTCTTATACACATCTTTAATTAA atctgaacagaaaagc3^′^	amplification of phosphorylated PhoZ transposon with mosiac end
Kan Prom TN5 F	5^′^P’CTGTCTCTTATACACATCTCCATGG taagataatatatc3^′^	amplification of phosphorylated PhoZ transposon with mosiac end
Ermx IL-10 homo F	5^′^ccagtagtactaaaagggctcGACGTCGCGGCCGCtta3^′^	homologous overlap of IL-10 gene cassette for OEP
Il10 Ermx homo R	5^′^taaGCGGCCGCGACGTCgagcccttttagtactactgg3^′^	homologous overlap of erythromycin resistance gene cassette for OEP
TN5 21 IL-10 F	5^′^P’CTGTCTCTTATACACATCTCCATGG taaatagagatctacagtatatggctaaatg3^′^	amplification of phosphorylated IL-10 transposon with mosiac end
Ermx TN5	5^′^P’CTGTCTCTTATACACATCTcggcggtggcatcattct3^′^	amplification of phosphorylated IL-10 transposon with mosiac end
Erm-hIL-10 BamHI F	5^′^cattagGGATCCCCATGGcggcggtggcatcattctggaaa3^′^	amplification of gblock containing erythromycin resistance and hIL-10 for plasmid insertion
HIL10-Erm EcoRI F	5^′^cattaggaattctaagataatatatcttttatatagaa gatatcgccgtatg3^′^	amplification of gblock containing erythromycin resistance and hIL-10 for plasmid insertion

**Table:** KEY RESOURCES TABLE

REAGENT or RESOURCE	SOURCE	IDENTIFIER

Antibodies		

Anti-human CD3 PECy7	BioLegend	RRID:AB_314052
Anti-human CD4 AF488	BioLegend	RRID:AB_389311
Anti-human CD45 BV510	BioLegend	RRID:AB_2687376
Anti-human CD69 PerCP/Cyanine5.5	BioLegend	RRID:AB_2074957
Anti-human CD8a BV421	BioLegend	RRID:AB_2629583
Anti-human gd TCR PE	BioLegend	RRID:AB_1089219
Anti-human Ki67 AF700	BioLegend	RRID:AB_2564039
Anti-human TCRbeta APC	BioLegend	RRID:AB_3662280
ARG-1 PerCP710	Invitrogen	RRID:AB_2734843
CD103 FITC	eBioscience	RRID:AB_10709438
CD103 PEDazzle 594	BioLegend	RRID:AB_2566492
CD11b PEDazzle 594	BioLegend	RRID:AB_2563647
CD11c BV421	BioLegend	RRID:AB_10897814
CD206 BV605	BioLegend	RRID:AB_2562340
CD4 BV510	BioLegend	RRID:AB_2800580
CD44 APC	BioLegend	RRID:AB_312962
CD44 BV510	BioLegend	RRID:AB_2561391
CD45 PerCP	BD Biosciences	RRID:AB_10584327
CD62L FITC	BD Biosciences	RRID:AB_10893197
CD68 BV785	BioLegend	RRID:AB_2860684
CD8 BV510	BD Biosciences	RRID:AB_2739908
CD90.2 BV605	BD Horizon	RRID:AB_2665477
F4/80 PECy7	BioLegend	RRID:AB_893490
F4/80 SparkYG593	BioLegend	RRID:AB_2894428
gd TCR PECy7	BioLegend	RRID:AB_11203530
gdTCR AF488	BioLegend	RRID:AB_2562770
IFNg PE	BD Biosciences	RRID:AB_10894592
IL-17 BV421	BioLegend	RRID:AB_10900442
IL-6 PE	BD Biosciences	RRID:AB_10895584
iNOS APC	Invitrogen	RRID:AB_2573244
Ki67 PerCP Cy5.5	BioLegend	RRID:AB_2910307
Ly6C APC Cy7	BioLegend	RRID:AB_10643867
Ly6C FITC	BD Pharmingen	RRID:AB_10584332
Ly6G BV650	BioLegend	RRID:AB_2565881
MHCII BV510	BioLegend	RRID:AB_2561397
TCRb APC Cy7	BioLegend	RRID:AB_893626
TNFa PECy7	BD Pharmingen	RRID:AB_396761
Vg2 APC	BioLegend	RRID:AB_10899574
Ultra-LEAFtrade Purified anti-mouse CD3	BioLegend	RRID:AB_2810313
Syrian Hamster Anti-CD28 Monoclonal Antibody, Unconjugated, Clone 37.51	BD Biosciences	RRID:AB_394764
InVivoMAb anti-mouse IL-10R (CD210)	BioXcell	RRID:AB_1107611

Bacterial and virus strains		

*Corynebacteria mastitidis AS1*	St. Leger et al.^26^	N/A
*Escherichia coli* Top 10	Invitrogen	C404050
*Pseudomonas aeruginosa* strain PAC	Laboratory of Robert Shanks	PMID: 11581056

Biological samples		

Human Peripheral Blood Mononuclear Cells, Frozen	StemCell Technologies	70025.1
Leukocyte Reduction System (LRS)	StemCell Technologies	200–0093

Chemicals, peptides, and recombinant proteins		

Cell Activation Cocktail (with Brefeldin A)	BioLegend	423303
CellTrace^™^ Violet Cell Proliferation Kit, for flow cytometry	Invitrogen	C34557
Ophthalmic Strips: BioGlo Ophthalmic Strip	MedLine	17238–900-11

Critical commercial assays		

IL-10 Mouse Uncoated ELISA Kit with Plates	Fisher Scientific	RRID:AB_2574996
ELISA MAXtrade Deluxe Set Mouse IFN-g	Biolegend	430804
ELISA MAXtrade Deluxe Set Mouse IL-17a	Biolegend	432504
ELISA MAXtrade Deluxe Set Human IL-17A	Biolegend	433914
ELISA MAXtrade Deluxe Set Human IFN-g	Biolegend	430104
ELISA MAX^™^ Deluxe Set Human IL-10	Biolegend	430604
1-Step^™^ PNPP Substrate Solution	ThermoFisher	37621
Dynabeads^™^ Human T-Activator CD3/CD28	Gibco	11161D

Deposited data		

Harmonized mIL-10/Erm resistance gene cassette	NCBI	PP471980
Data used to create figures and graphs	Mendeley Data	10.17632/kwhys2zfwd.1

Experimental models: Cell lines		

Human Cornea Limbal Epithelial cells	Laboratory of Robert Shanks	RRID:CVCL_UZ28

Experimental models: Organisms/strains		

C57BL/6 mice	Jackson Laboratories	RRID:IMSR_JAX:000664

Oligonucleotides		

Primers used in this study, see Table 1	IDT	This manuscript
Harmonized mIL-10 g-block	IDT	This manuscript
Harmonized hIL-10 g-block	IDT	This manuscript
Harmonized Erythromycin resistance g-block	IDT	This manuscript

Recombinant DNA		

phosphatase Z gene from *Enterococcus faecalis*	Laboratory of Daria Van Tyne	N/A
Plasmid: pTony3	Rigas et al.^27^	N/A

Software and algorithms		

FIJI	Schindelin et al.^37^	https://imagej.net/software/fiji/
Codon Harmonizer	Claassens et al.^38^	https://codonharmonizer.systemsbiology.nl/
SignalP 5.0	Almagro et al.^29^	https://services.healthtech.dtu.dk/services/SignalP-5.0/

## Highlights

- Eye-colonizing bacteria are genetically engineered to colonize the eye while secreting IL-10
- Microbial-derived IL-10 can suppress the production of inflammatory factors *in vitro*
- Microbial-produced and secreted IL-10 accelerates corneal epithelial wound repair
