# Supplementary material for: Genetically engineered eye-colonizing microbes that deliver the anti-inflammatory cytokine interleukin-10 enhance corneal tissue repair
Source: Cell Rep. Author manuscript; Available in PMC 2026 Jul 28. (PMC13411304; doi:10.1016/j.celrep.2026.117064)
Supplement: 1 [file NIHMS2160130-supplement-1.pdf]

Cell Reports, Volume *45*

Supplemental information

Genetically engineered eye-colonizing microbes that deliver the anti-inflammatory cytokine interleukin-10 enhance corneal tissue repair

Jackie Shane, Matthew Evans, Yannis Rigas, Robert M.Q. Shanks, and Anthony J. St. Leger

Table S1. **mIL-10 in tears of inoculated mice**

mIL-10 (pg/mL)			
PBS (-)	AS1	mIL10.4	mIL10.14
16.43954	20.6217	35.09792	11.41867
35.2436	40.33942	19.16888	13.15003
11.72275	46.45497	25.70447	9.269437
33.58888	19.90926	24.60643	11.677
26.16277	15.54003	19.98019	23.09588
4.817325	2.622214	5.323091	12.63276
4.46495	4.131625	12.22937	2.174016
4.841083	10.25471	4.628451	8.956763
5.717836	5.02032	24.58761	4.007121
5.717836	7.467016	3.80573	12.8985

**Table S2. Antibody Panels**				
Fig.	Surface/Intra	Antibody	Clone	Vendor
2b	Surface	TCRb APC Cy7	H57-597	BioLegend
	Surface	gd TCR PECy7	GL3	BioLegend
	Surface	CD8 BV510	53-6.7	BD Biosciences
	Surface	CD4 BV510	RM4-5	Pac Blue
	Surface	CD44 APC	IM7	BioLegend
	Surface	CD62L FITC	MEL-14	BD Biosciences
	Intracellular	Ki67 PerCP Cy5.5	16A8	BioLegend
3d-g	Surface	TCRb APC Cy7	H57-597	BioLegend
	Surface	CD44 BV510	IM7	BioLegend
	Surface	gdTCR AF488	GL3	BioLegend
	Surface	Vg2 APC	UC3-10A6	BioLegend
	Surface	CD90.2 BV605	53-2.1	BD Horizon
	Surface	CD103 PEDazzle 594	2E7	BioLegend
	Intracellular	TNFa PECy7	MP6-XT22	BD Pharmingen
	Intracellular	IL-17 BV421	TC11-18H10.1	BioLegend
	Intracellular	IFNg PE	XMG1.2	BD Biosciences
3h	Surface	CD45 PerCP		

	Surface	CD11b PEDazzle 594	M1/70	BioLegend
	Surface	CD11c BV421	N418	BioLegend
	Surface	Ly6G BV650	1A8	BioLegend
	Surface	MHCII BV510	M5/114.15.2	BioLegend
	Surface	F4/80 PECy7	BM8	BioLegend
	Surface	Ly6C APC Cy7	HK1.4	BioLegend
	Surface	CD103 FITC	2E7	eBioscience
5e&f	Surface	CD11b PEDazzle 594	M1/70	BioLegend
	Surface	CD11c BV421	N418	BioLegend
	Surface	Ly6G BV650	1A8	BioLegend
	Surface	Ly6C FITC	AL-21	BD Pharmingen
	Surface	MHCII BV510	M5/114.15.2	BioLegend
	Surface	CD206 BV605	C068C2	BioLegend
	Surface	CD45 PerCP	30F11	BD Biosciences
	Intracellular	TNF PECy7	MP6-XT22	BD Pharmingen
	Intracellular	CD68 BV785	FA-11	BioLegend
	Intracellular	IL-6 PE		BD Biosciences
	Intracellular	iNOS APC	CXNFT	Invitrogen
	Intracellular	ARG-1 PerCP710	A1exF5	Invitrogen
5i	Surface	CD11b PEDazzle 594	M1/70	BioLegend
	Surface	CD11c BV421	N418	BioLegend
	Surface	Ly6G BV650	1A8	BioLegend
	Surface	Ly6C FITC	AL-21	BD Pharmingen
	Surface	MHCII BV510	M5/114.15.2	BioLegend
	Surface	CD206 BV605	C068C2	BioLegend
	Surface	CD45 PerCP	30F11	BD Biosciences
	Surface	F4/80 SparkYG593	QA17A29	BioLegend
	Intracellular	TNF PECy7	MP6-XT22	BD Pharmingen
	Intracellular	CD68 BV785	FA-11	BioLegend
	Intracellular	IL-6 PE		BD Biosciences
	Intracellular	iNOS APC	CXNFT	Invitrogen
	Intracellular	ARG-1 PerCP710	A1exF5	Invitrogen
6b-d	Surface	Anti-human CD3 PECy7	UCHT1	BioLegend
	Surface	Anti-human CD45 BV510	HI30	BioLegend
	Surface	Anti-human CD8a BV421	RPA-T8	BioLegend

	Surface	Anti-human CD4 AF488	RPA-T4	BioLegend
	Surface	Anti-human gd TCR PE	B1	BioLegend
	Surface	Anti-human TCRbeta APC	IP26	BioLegend
	Surface	Anti-human CD69 PerCP/Cyanine5.5	FN50	BioLegend
	Intracellular	Anti-human Ki67 AF700	Ki67	BioLegend
